# The transcription factor Ndt80 is a repressor of Candida parapsilosis virulence attributes

**DOI:** 10.1080/21505594.2021.1878743

**Published:** 2021-02-04

**Authors:** Joana Branco, Cláudia Martins-Cruz, Lisa Rodrigues, Raquel M. Silva, Nuno Araújo-Gomes, Teresa Gonçalves, Isabel M. Miranda, Acácio G. Rodrigues

**Affiliations:** a Division of Microbiology, Department of Pathology, Faculty of Medicine, University of Porto, Porto, Portugal; b CINTESIS - Center for Health Technology and Services Research, Faculty of Medicine, University of Porto, Porto, Portugal; c CNC - Centre for Neuroscience and Cell Biology, University of Coimbra, Coimbra, Portugal; d FMUC - Faculty of Medicine, University of Coimbra, Coimbra, Portugal; e Faculdade De Medicina Dentária, CIIS - Centro De Investigação Interdisciplinar Em Saúde, Universidade Católica Portuguesa, Viseu, Portugal; f Cardiovascular R&D Centre, Faculty of Medicine, University of Porto, Porto, Portugal

**Keywords:** *Candida parapsilosis*, transcription factor, fungal morphogenesis, fungal adhesion, biofilm, Als-like, immune system evasion, macrophage phagocytosis, invasive fungal infection

## Abstract

*Candida parapsilosis* is an emergent opportunistic yeast among hospital settings that affects mainly neonates and immunocompromised patients. Its most remarkable virulence traits are the ability to adhere to prosthetic materials, as well as the formation of biofilm on abiotic surfaces. The Ndt80 transcription factor was identified as one of the regulators of biofilm formation by *C. parapsilosis*; however, its function in this process was not yet clarified. By knocking out *NDT80* (*CPAR2-213640*) gene, or even just one single copy of the gene, we observed substantial alterations of virulence attributes, including morphogenetic changes, adhesion and biofilm growth profiles. Both *ndt80Δ* and *ndt80ΔΔ* mutants changed colony and cell morphologies from smooth, yeast-shaped to crepe and pseudohyphal elongated forms, exhibiting promoted adherence to polystyrene microspheres and notably, forming a higher amount of biofilm compared to wild-type strain. Interestingly, we identified transcription factors Ume6, Cph2, Cwh41, Ace2, Bcr1, protein kinase Mkc1 and adhesin Als7 to be under Ndt80 negative regulation, partially explaining the phenotypes displayed by the *ndt80ΔΔ* mutant. Furthermore, *ndt80ΔΔ* pseudohyphae adhered more rapidly and were more resistant to murine macrophage attack, becoming deleterious to such cells after phagocytosis. Unexpectedly, our findings provide the first evidence for a direct role of Ndt80 as a repressor of *C. parapsilosis* virulence attributes. This finding shows that *C. parapsilosis* Ndt80 functionally diverges from its homolog in the close related fungal pathogen *C. albicans*.

## Introduction

*Candida parapsilosis* is a ubiquitous yeast, often recovered from domestic animals, soil, and marine environments, but is also a commensal of the human skin. Among hospital settings, this species is considered a major opportunistic pathogen involved in invasive fungal infections [1,2]. Its incidence has dramatically increased, being the second most common *Candida* species isolated from blood cultures in Latin America, Asia, and Southern Europe countries [3–8]. *C. parapsilosis* is of particular concern among susceptible populations, comprising low birth weight neonates, immunocompromised individuals, and patients requiring prolonged use of indwelling devices such as central venous catheters [1,9]. Besides its ability to grow and persist in the hospital environment surfaces, *C. parapsilosis* stands out for its capacity to adhere to the abiotic surface of implanted devices, later involving biofilm formation [1,10,11]. In fact, adhesion and formation of biofilm are intimately related with *C. parapsilosis* virulence and are critical for its involvement in hospital outbreaks [2].

Biofilm is an organized community comprised of a dense network of microbial cells embedded in an extracellular matrix of polymers, which clinically restricts drug access and the immune response [12,13]. The ability of fungal cells to adhere to host tissues or medical indwelling devices, as well as cell-cell binding are required for biofilm development and for infection proliferation [14–16]. In contrast to *C. albicans, C. parapsilosis* does not form true hyphae and, therefore, its biofilm only involves yeast and pseudohyphal forms [17,18]. To identify putative *C. parapsilosis* biofilm regulators, more than 100 transcription factors were knocked-out and mutants were assessed for biofilm formation ability [19]. Previously identified as biofilm regulators in *C. albicans*, Bcr1, Efg1 and Ace2 were also directly implicated in biofilm development in *C. parapsilosis* [16,19–22], together with the transcription factor Gzf3, whose involvement in biofilm formation seems to be restricted to *C. parapsilosis* [19]. In this large-scale screen of *C. parapsilosis* biofilm defective mutants, *NDT80* was firstly pointed as a putative biofilm regulator, in analogy with *C. albicans* biofilm regulation network. However, in the case of *C. parapsilosis, NDT80* role was undisclosed due to marked growth defects exhibited by *ndt80* mutant [19]. In *C. albicans*, Ndt80 was first described as a key modulator of azole drug sensitivity, being involved in the control of ergosterol biosynthesis [23] and activation of the efflux pump Cdr1 [24]. We firstly identified *C. parapsilosis* Ndt80 ortholog to be a transcription factor upregulated following azole resistance acquisition [25]. Later, we showed that *ndt80* mutant exhibits increased susceptibility to azoles and that, together with Upc2 transcription factor, also regulates the expression of various genes of ergosterol biosynthetic pathway, namely *ERG25, ERG6, ERG2, ERG3* and *ERG4* [26].

In this study, we address the role of Ndt80 in *C. parapsilosis* as a repressor of virulence attribute expression, namely morphogenesis, adhesion, and biofilm formation. Additionally, we explore the morphological phenotypes, its constitutive filamentous growth, and the adhesion profile resulting from *NDT80* knockout, as well as its interaction with host immune system by assessing macrophage-mediated response.

## Methods

### Culture conditions

Yeast strains used in this study were routinely grown in YPD broth medium (1% yeast extract, 2% bacto-peptone, 2% glucose) at 30°C with agitation (180 rpm) or on YPD agar plates, following addition of 2% of agar. To recycle the *SAT1* flipper cassette, transformants were incubated in YPM medium (1% yeast extract, 2% peptone, 2% maltose) overnight, with agitation (180 rpm); afterward, approximately 100 cells were plated on YPD plates supplemented with nourseothricin at final concentration of 20 μg ml^−1^. All *C. parapsilosis* strains were stored in YPD broth with 40% glycerol, at – 80°C.

RAW 264.7 murine macrophages were obtained from the European Collection of Cell Cultures and maintained in DMEM (Sigma-Aldrich) with 10% non-inactivated Fetal Calf Serum (FCS), 10 mM HEPES, 12 mM sodium bicarbonate and 11 mg ml^−1^ sodium pyruvate at 37°C in a humidified atmosphere with 5% CO_2_. The culture medium was changed every 2 days, until ~70% of cell confluence was reached. RAW 264.7 cells were resuspended in RPMI 1640 medium (Sigma-Aldrich) supplemented with 10% inactivated FCS, 23.8 mM sodium bicarbonate and 50 mM glucose for the experimental assays (initiated until the cells 15^th^ generation).

### Plasmid construction

To knockout *NDT80* gene in *C. parapsilosis* BC014S (wild-type strain) [25], the pNG4 disruption cassette described by Branco *et al*. [26] was used. Briefly, a 478 bp upstream and 460 bp downstream sequences of *NDT80* gene were amplified using CpNDT80up_F and CpNDT80up_R primers (containing recognition sites for *KpnI* and *ApaI*) and CpNDT80down_F and CpNDT80down_R primers (containing recognition sites for *SacII* and *SacI*), respectively, and cloned into the flanking sites of pCD8 plasmid [18]. After restriction with *KpnI* and *SacI*, pNG4 disruption cassette was introduced into the native locus of *NDT80* gene of *C. parapsilosis* BC014S. All primer sequences are listed in Table 1.

**Table 1.** Primers used in this study

Primer name	Primer sequence (5` to 3`)
Construction of deletion cassette	
CpNDT80up_F	GGGGGTACCGGCAATTTTGATTTTTGGGTTC
CpNDT80up_R	GGGGGGCCCGAGGCACCACCAGCAGTAGAGT
CpNDT80down_F	TCCCCGCGGGATGGGAGAAAAAACTGAACCTTG
CpNDT80down_R	CGAGCTCAGATGGCATTGTAGTCAGTAGCATC
PCR Confirmation	
CpNDT80gen_F	GCCTTTTACATCTATCGAAGTCAAACTTG
FLP_R	TTTATGATGGAATGAATGGGATG
RT-qPCR	
CpACT1_F1	TGCTCCAGAAGAACACCCA
CpACT1_R1	CACCTGAATCCAAAACAATACCAGT
CpBCR1_F	TCGCCACCACTACTCG
CpBCR1_R	AAAGGATAATGTTGCTGTGA
CpEFG1_F	GAGCGGAGCAGCAGTT
CpEFG1_R	GAAGCATAAGGTTGTTGGG
CpACE2_F	AACAACAACAACAACCCC
CpACE2_R	ACATCTAAATCCTGCAATCC
CpUME6_F	CTTTTCCCCCGTCTGTA
CpUME6_R	TGCAATGTTTTCTGTTCACT
CpMKC1_F	TCAGAGAATCCAGAACAAAA
CpMKC1_R	ATCCAACAGACCACACG
CpCZF1_F	CCAACAACAAAACTCCAAC
CpCZF1_R	TCTCGACTCACAACATCTCT
CpGZF3_F	GATACATTCAAAGCAGCAAA
CpGZF3_R	GTGGTTATCTTCAGTTCCG
CpCPH2_F	TCCAAAGTGACAAAGCC
CpCPH2_R	GCAATTCTCAAAGCAGG
CpRHR2_F	TTTGTTTGACTGTGACGG
CpRHR2_R	TACGGCATCCATGAGAAG
CpALS3_F	CGCACCAGCAAACTCATCAA
CpALS3_R	CCAATGAACTCGGGGGAAAT
CpALS7_F1	CTTCTGTTGTTGTGTCATCCCTG
CpALS7_R1	CACCATCTGTTGAGCCTGTAG
NDT80_F3	CAAAGGGCGGTATGAATGGTA
NDT80_R3	TGGTGTGGATGGTGTGGA
CpCW41_F	TGACGACGACGATGAACGCG
CpCW41_R	TGGTGATGAGCGGGGATA
CpSTP3_F	TCCGCCACGATAAAGCCA
CpSTP3_R	GAATCACCCAGACCACCG
CpOCH1_F	AATGCGATGCCCTTGTTGC
CpOCH1_R	TTGCTTGCCCACTCGTCA

### C. parapsilosis *transformation*

Transformation of wild-type strain was performed by electroporation as described by Ding *et al*. [18]. Briefly, an overnight cell culture was diluted in 50 ml of YPD broth medium for an initial OD_600_ of 0.2 and incubated at 30°C until reaching approximately OD_600_ of 2.0. After being pelleted, yeast cells were resuspended in 10 ml of Tris-EDTA buffer (10 mM Tris-HCl, 1 mM EDTA, pH 7.5) containing 10 mM dithiothreitol and incubated at 30°C for 1 h with agitation (100 rpm). Yeast cells were washed twice with 40 ml of cold water plus once with 10 ml 1 M Sorbitol and, subsequently resuspended in 125 μl of this solution. Approximately 1 µg of purified *KpnI-SacI* fragment of pNG4 was added to 50 µl of competent cells. The cell mixture was then transferred to a 1 mm electroporation cuvette. Electroporation shock was performed at 1.25 kV, using a Gene Pulser X-cell Electroporator (Bio-Rad). Afterward, 950 μl of YPD containing 1 M sorbitol was immediately added; the mixture was incubated at 30°C for 4 h with agitation; afterward 100 μl were plated on YPD agar supplemented with nourseothricin at final concentration of 200 μg ml^−1^. Transformants were obtained after 24 h of incubation at 30°C.

### Adhesion assay

Yeast adhesion was quantified by flow cytometry, as described by Silva-Dias *et al*. [27]. Briefly, yeasts were grown overnight at 30°C in Sabouraud broth medium, with agitation (180 rpm); the culture was centrifuged at 10,000 *g* for 5 min and washed twice with phosphate buffer saline (PBS) (Sigma-Aldrich). A yeast suspension was standardized to 2 × 10^6^ cells ml^−1^ in the same buffer and mixed with 2 × 10^7^ microspheres ml^−1^ of 1 μm uncoated carboxylated highly green fluorescent polystyrene microspheres (Molecular Probes). This mixture was incubated at room temperature for 30 min at 150 rpm. The suspensions were vortexed, and 50,000 events were analyzed using a FACS Calibur flow cytometer (BD Biosciences). Cell adhesion results are expressed as the percentage of cells with microspheres attached, representative of at least three independent experiments, performed in triplicate.

### Biofilm formation assays

After overnight growth at 37°C with agitation (180 rpm) in Sabouraud broth medium, yeast cells were collected by centrifugation at 10,000 *g* for 5 min, washed once with PBS and standardized to obtain a suspension of 1 × 10^6^ yeast cells ml^−1^ in RPMI-1640 medium supplemented with L-glutamine and buffered with MOPS acid (Sigma-Aldrich). One ml of such cell suspension was placed in each of a 12-well polystyrene microplate and incubated for 24 and 48 h at 37°C. Following incubation, total biomass was quantified by Crystal Violet (CV) assay, as previously described by Silva-Dias *et al*. [28]. Biofilm mass was calculated from at least three independent experiments, performed in triplicate.

For dry mass assessment, *C. parapsilosis* strains were set up as previously described, except the standardization of the cell suspension, which was diluted to an OD_600_ of 1; afterward, 5 ml were distributed in each well of a 6-well polystyrene plate. After 24 and 48 h of incubation at 37°C, adherent biofilms were washed with PBS, scrapped from the bottom of the wells, and vacuum filtered, as described by Holland, *et al*. [19]. The average of the total biomass was calculated by subtracting the initial weight of the filter to the final weight, determined from three independent experiments, performed in triplicate.

### Microscopic imaging

Colony phenotypes were observed and photographed under 20× magnification using a Stereo zoom S9i (Leica Microsystems) dissection microscope, after growth on YPD agar at 30°C, for 72 h. Images of yeast cell morphology were taken with a Zeiss Axioplan microscope, coupled with an AxioVision image acquisition system (Zeiss), after staining with Calcofluor White (Sigma-Aldrich) and mounting on glass slides. Yeast cells were photographed under 1000× magnification, oil immersion.

### RNA extraction, cDNA synthesis and RT-qPCR

RNA was extracted as described by Kohrer and Domdey [29]. Concentration and quality of RNA samples were measured using a Nanodrop equipment (Eppendorf). Only samples yielding A_280_/A_260_ ratios ranging from 1.6 to 2.2 and showing no signs of degradation, after electrophoresis, were used in subsequent analyses.

From 100 ng of total RNA, the first-strand cDNA was synthesized using the SensiFAST cDNA Synthesis Kit (Bioline) according to the manufacturer´s instructions. The resulting cDNA was stored at −20°C prior to use for real-time quantitative polymerase chain reaction (RT-qPCR). The genes analyzed were the followed: *NDT80* (*CPAR2_213640*), *OCH1* (*CPAR2_404930*), *ALS3* (*CPAR2_404770*), *ALS7* (*CPAR2_404800*), *GZF3* (*CPAR2_800210*), *ALS7* (*CPAR2_404800*), *BCR1* (*CPAR2_205990*), *EFG1* (*CPAR2*_*701620*), and the orthologues of *Candida albicans STP3* (*CPAR2_200390*), *CWH41* (*CPAR2_501400*), *STP3* (*CPAR2_200390*), *MKC1* (*CPAR2_800090*), *CPH2* (*CPAR2_603440*), *RHR2* (*CPAR2_503990*), *ACE2* (*CPAR2_204370*), *CPH2* (*CPAR2_603440*), *UME6* (*CPAR2_803820*) and *CZF1* (*CPAR2_501290*).

For each real-time quantitative PCR, five replicates per strain were analyzed. All primers used are detailed in Table 1. PCRs were performed using the SensiFAST SYBR Hi-ROX Kit (Bioline) 3-step cycling, according to the manufacturer’s instructions, in a PikoReal Real-Time PCR System instrument (Thermo Scientific). *ACT1* gene expression was used to normalize the signal obtained for each gene. Data obtained were analyzed with REST software.

### Bioinformatic analysis

Sequences from *C. parapsilosis* CDC317 open reading frames (ORFs) plus 1000 bp upstream and downstream (version s01-m03-r14, from 7 February 2016) were downloaded from the Candida Genome Database (CGD, http://candidagenome.org/). To identify putative Ndt80-regulated genes, a search for the MSE consensus motif (gNCRCAAAY) was performed in the promoter regions (1000 bp upstream the start codon). The resulting ORFs containing MSE sequences were grouped according to Gene Ontology (GO) terms using the CGD Gene Ontology Slim Mapper with the default parameters.

### Macrophage-yeast interaction assays

Macrophage-yeast interaction assays were carried out as previously described [30]. Briefly, RAW 264.7 macrophage cells were platted in 96-, in 12-well (with 16 mm glass coverslips) or in µ-slide 8 well plates, and incubated for 18 h at 37°C, under a 5% CO2 atmosphere. After this incubation period, yeast cells were added to the macrophages at an MOI (Multiplicity of Infection) of 1:1.

#### Immunofluorescence and microscopic analysis

Macrophages grown in coverslips were incubated with *C. parapsilosis* as described below. At the end of each incubation period (10 min, 30 min, 1 h 30 min, 3 h), coverslips were washed twice with ice-cold PBS and fixed with 4% paraformaldehyde in PBS for 15 min at room temperature. After 3 washing steps with PBS, cell membranes were stained with WGA, for 10 min, protected from light. Macrophages were treated with a blocking solution of 10% bovine serum albumin in PBS for 30 min at 37°C. Cells were then incubated overnight, at room temperature, with the primary rabbit polyclonal antibody against Candida (GTX40096; GeneTex), diluted (1:200) in blocking solution. Coverslips were washed and incubated for 2 h at room temperature with the AlexaFluor 488 donkey anti-rabbit IgG secondary antibody (A21206; Invitrogen). Finally, after a washing step, macrophage cells were incubated with DAPI 0.02% for 10 min at room temperature. Cells were subsequently washed and the coverslips were mounted in glass slides with DAKO mounting medium and kept at −20°C until observation under confocal or fluorescence microscopy. Digital images were captured using a Carl Zeiss LSM 710 Confocal Microscope, using Plan-ApoChromat 40x/63x/1.4 oil objectives; Zen Blue and FiJi software’s were used to analyze the images.

#### Yeast and macrophage viability assays

The yeast cell viability following interaction with RAW 264.7 macrophage cells was assessed by a colony-forming unit (CFU) assay. After 30 min and 3 h of co-incubation, supernatants were collected and plated on YPD agar, to count non-internalized or non-adhered yeast cells. The remaining adhered RAW 264.7 macrophages were scraped and lysed with 0.5% Triton X-100. This cell suspension, representing the amount of yeast cells internalized was plated on YPD agar, using serial dilutions. Following 3 days of incubation, at 30°C, the number of yeast colonies per ml was calculated.

For macrophage viability assay, after 30 min and 3 h of co-incubation, viable, and death macrophage cells were calculated using a hemocytometer, after staining with Trypan Blue (T8154; Sigma-Aldrich).

#### Live cell imaging assays

For live cell imaging assays, culture media without phenol-red was used and macrophage cell membranes were stained with Wheat Germ Agglutinin, Tetramethylrhodamine conjugate (WGA, W849; Molecular Probes). Image acquisitions were conducted during at least 45 min, using a confocal Cell Observer Spinning Disk microscope (Zeiss), equipped with an LCI PlanNeofluar 63x/1.3 glycerol objective; Zen Blue software was used to analyze the time-lapse videos obtained.

### Statistical analysis

Statistical analysis of results of adhesion, biofilm and infection assays was performed using one-way ANOVA followed by a Dunnett post hoc test. Differences were considered statistically significant for a p-value <0.05. Significant differences were marked with an asterisk character (*), in which *p < 0.05, **p < 0.01, ***p < 0.001. All results are presented as mean ± standard deviation, of at least three independent experiments.

## Results

### *Deleting* NDT80 *transcription factor gene triggers morphogenesis*

**Figure 1.**
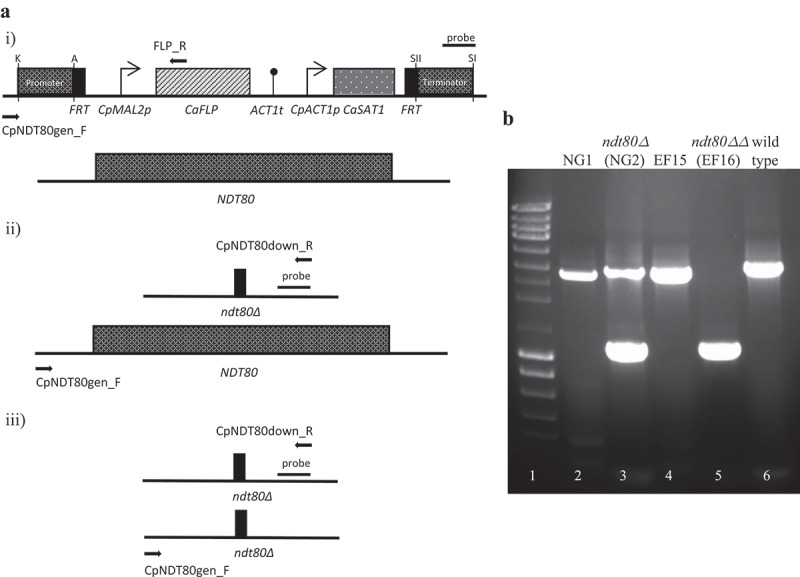
Deletion of *NDT80* transcription factor gene in *C. parapsilosis*. Gene knockout was confirmed by PCR. Genomic integration of *NDT80* disruption cassette in the wild-type strain was confirmed using the following pairs of primers CpNDT80gen_F and FLP_R (a, i), which amplified a 2.9 kb fragment (b, NG1 strain, lane 2). The recycling of the disruption cassette was confirmed using primers CpNDT80gen_F and CpNDT80down_R (a, ii), originating a 3.1 kb (second copy of *NDT80* gene) and 1.2 kb PCR products (disruption of the first copy) (b, NG2 strain, lane 3). Disruption of the second allele in strain NG2 was confirmed following the same strategy, using the primers: CpNDT80gen_F and FLP_R (a, i), which amplified a 2.9 kb fragment that corresponds to the second integration of *NDT80* disruption cassette (b, EF15 strain, lane 4) and CpNDT80gen_F and CpNDT80down_R (A, iii), amplifying a 1.2 kb PCR product, indicating a successful recycling of the cassette (b, EF16 strain, lane 5). Wild-type strain was used as PCR control of CpNDT80gen_F and CpNDT80down_R pair primers, amplifying a 3.1 kb fragment (b, lane 6). Lane 1 represents the molecular size marker (NZYDNA Ladder III, NZYTech)

To gain insight into the role of Ndt80 in *C. parapsilosis* virulence attribute expression, two independent lineages lacking one (*ndt80Δ* – NG2 strain) or both (*ndt80ΔΔ* – EF16 strain) copies of *NDT80* were generated from *C. parapsilosis* strain BC014S (wild-type strain) [25]. Deletion was carried out using a gene-specific disruption cassette (pNG4) based on the recyclable nourseothricin-resistant marker as previously described [18]. The introduction of pNG4 into the*NDT80* locus of the wild-type strain, generated NG1 clone, which after cassette recycling, resulted in the NG2 strain. To delete the second copy of *NDT80* gene, a second round of integration/recycling were performed, generating EF15 and EF16 clones, respectively. Gene knockout was confirmed by PCR (Figure 1(a) and (b)).

**Figure 2.**
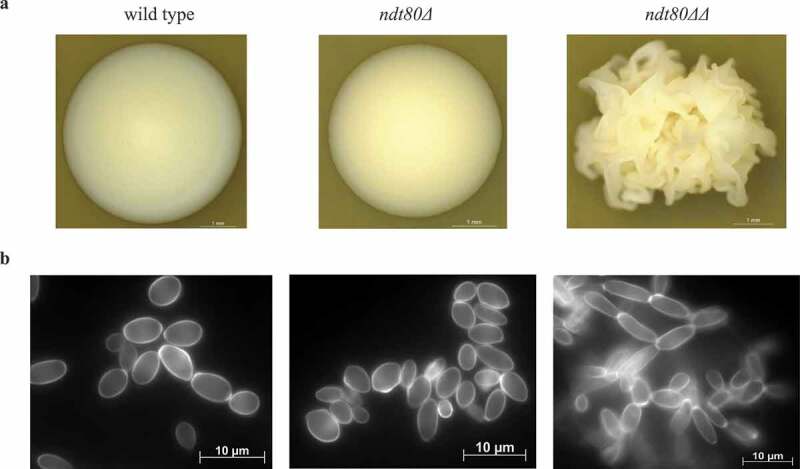
*NDT80* deletion triggers morphogenesis changes in *C. parapsilosis*. (a) Colony morphology of wild-type, *ndt80Δ* and *ndt80ΔΔ* strains. Yeasts were grown at 30°C for 2 days and colonies photographed under 20× magnification. Smooth colonies were found in wild-type strain; *ndt80Δ* mutant displays creaky-opaque colonies, while only crepe phenotype colonies were observed in the *ndt80ΔΔ* mutant strain. (b) Cell morphology of wild-type, *ndt80Δ* and n*dt80ΔΔ* strains. Staining of wild-type and *ndt80Δ* cells with calcofluor white revealed a cell population mainly composed by yeasts; in contrast, *ndt80ΔΔ* mutant shows a mixture of elongated cells and pseudohyphae. Cells were visualized under fluorescence microscopy and photographed under 1000× magnification, oil immersion

Deletion of *NDT80* had a major effect upon colony and yeast cell morphology (Figure 2(a) and (b)). The parental strain and the *ndt80Δ* haploid mutant grow as smooth-white and creaky-opaque colonies, respectively, whereas colonies from *ndt80ΔΔ* diploid mutant display a crepe phenotype. Wild-type and haploid cells are yeast-shaped cells; in contrast, the *ndt80ΔΔ* cell population is mostly composed of elongated cells and pseudohyphae.

### *Deleting* NDT80 *increases adhesion and biofilm formation ability*

**Figure 3.**
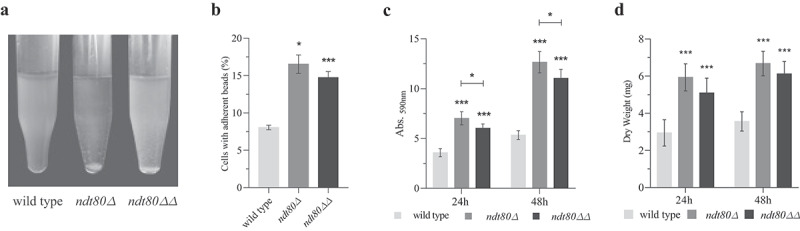
Deletion of *NDT80* increases adherence and biofilm formation ability. (a) Images of wild-type, *ndt80Δ* and *ndt80ΔΔ* strains grown in liquid media; the mutants strains exhibit a strong flocculation (cell-cell adhesion) phenotype. (b) Percentage of yeast cells with adherent beads. *ndt80Δ* and *ndt8ΔΔ* mutants exhibited significantly higher adhesion ability than wild-type. The ability to form biofilm was quantified by (c) Cristal Violet (CV) staining and (d) dry weight, following 24 and 48 h of growth; in both assays, a significant increase of biofilm formation by *ndt80Δ* and *ndt8ΔΔ* mutants compared to the parental strain was observed. CV staining revealed a statistical decrease in biofilm formation between *ndt80Δ* and *ndt8ΔΔ* mutants, at both time points. *p < 0.05, **p < 0.01 and ***p < 0.001 wild-type vs *ndt80Δ* and *ndt8ΔΔ* mutants, or both groups

The yeast to pseudohyphae transition was observed along with the formation of fungal cell aggregates, typical of enhanced cell to cell adhesion. The *ndt80Δ* and *ndt80ΔΔ* mutants flocculate in liquid medium, suggesting that Ndt80 negatively affects the cell-cell adhesion process (Figure 3(a)). The ability of *C. parapsilosis* to adhere to polystyrene microspheres, representative of abiotic surfaces, was quantified using a flow cytometric adhesion assay, as described previously [27]. Compared to wild-type, manipulated strains displayed a significant increase of about 2-fold in adhesion ability (Figure 3(b)).

Filamentous growth and adhesion displayed by *ndt80ΔΔ* mutant are two known enhancers of biofilm formation. We assessed wild-type and mutants strains regarding the ability to form biofilm, using two independent methods, Cristal Violet (CV) staining [28] and dry weight [19]. *C. parapsilosis* lacking one or both copies of *NDT80* gene exhibits enhanced capacity to form biofilm compared to wild-type strain (Figure 3(c) and (d)). Differences were statistically significant when using both methodologies. Nevertheless, comparatively to *ndt80Δ* mutant, *ndt80ΔΔ* mutant had lower biofilm biomass, a result statistically significant when using CV staining for biofilm quantification.

### Ndt80 regulates the expression of adhesion-, morphology- and biofilm-related genes

**Figure 4.**
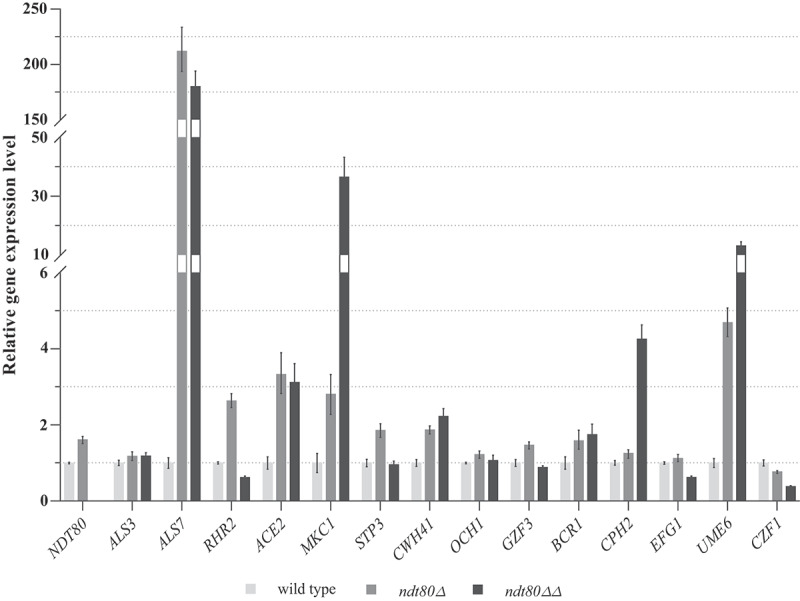
Putative targets of Ndt80 transcription factor. Relative expression levels of *NDT80, ALS7, ALS3, CZF1, UME6, GZF3, CPH2, EFG1, BCR1, ACE2, STP3, CWH41, OCH1, RHR2* and *MKC1* genes in *ndt80Δ* and *ndt8ΔΔ* strains compared with wild-type strain. *ACT1* was used as a normalizer gene. Expression values represent the mean value and ± standard deviation of five independent experiments

A set of transcription factor genes, namely Czf1, Ume6, Gzf3, Cph2, Efg1, Bcr1, Ace2, additional regulators like Stp3, Cwh41, Och1, Rhr2, one protein kinase (Mkc1) and also adhesins Als-like (Als7, Als3), were identified by several authors [19,31,32] as regulators of morphology transition, and as effectors in adhesion and biofilm formation by *C. parapsilosis*. In an attempt to identified Ndt80 targets involved in triggering virulence factors, we quantified the expression of the above-mentioned genes by RT-qPCR (Figure 4).

Relatively to adhesin-like genes, the expression of *ALS7* in *ndt80Δ* and *ndt80ΔΔ* mutants was upregulated 210- and 180-fold, respectively, compared to wild-type. In contrast, *ALS3* gene expression was not changed significantly among the studied mutant strains. The expression of *UME6* was upregulated, approximately, 5-fold in the *ndt80Δ* haploid mutant and a 13-fold in the *ndt80ΔΔ* diploid mutant, compared to the wild-type. *MKC1* expression was also upregulated 2.8-fold and 36-fold in haploid and diploid mutants, respectively, comparatively to the wild-type. *CPH2* gene exhibited a 1.2-fold upregulation in *ndt80Δ* mutant and of approximately 4-fold increased expression in *ndt80ΔΔ* mutant, in comparison to the wild-type.

*ACE2, CWH41* and *OCH1* genes displayed similar expression values of approximately 3-fold, 2-fold, and 1.2-fold, respectively, in the haploid and diploid mutants. *BCR1* gene was 1.5 and 1.7-fold upregulated in *ndt80Δ* and *ndt80ΔΔ* mutants in comparison to wild-type. The expression of *STP3* was increased approximately 1.8-fold in *ndt80Δ* mutant but remained unchanged in *ndt80ΔΔ* mutant. In contrast, *EFG1, GZF3* and *RHR2* were downregulated in *ndt80ΔΔ* mutant comparatively to the wild-type; *ndt80Δ* mutant exhibited a slight upregulation of expression of such genes (of about 1.1-, 1.4-, and 2.6- fold, respectively). *CZF1* gene was progressively downregulated following sequential *NDT80* gene copy deletion, by approximately 30% and 70%, respectively.

As expected, no *NDT80* transcript was observed in the null strain. Interestingly, the expression of *NDT80* in *ndt80Δ* mutant was 1.6-fold up-regulated. Since *NDT80* gene has in its promoter region the MSE binding sequence, we could hypothesize that to cope with one copy gene deletion, Ndt80 up-regulates itself expression, as described in *S. cerevisiae* and *A. nidulans* [33,34].

### *Identification of putative* NDT80*-regulated genes*

Ndt80 was found to bind to the middle sporulation element (MSE) (5ʹ-CACAAA-3ʹ) in the target gene promoter region [35] of *C. albicans* and *S. cerevisiae* ORFeomes [22,23]. The putative colony transition, adhesion- and biofilm-related genes mentioned above were analyzed for the presence of MSE motifs using the NCBI blast tool. As some of the promoter regions bound by biofilm regulators are larger than the normal [22,35], the considered sequence was approximately 1 kb upstream of the start codon. All genes assessed for their expression (Figure 4) contain putative MSE recognition sites, being identified in promoter regions.

**Figure 5.**
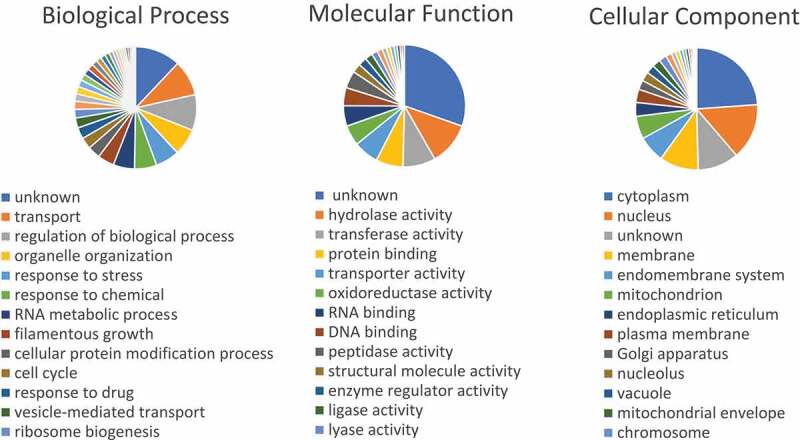
GO analysis of *Candida parapsilosis* genes putatively regulated by the Ndt80 transcription factor. ORFs containing MSE elements are grouped according to Biological Process, Molecular Function and Cellular Component

Attaching to such results, we further expanded the search for MSE consensus sequences in the complete *C. parapsilosis* ORFeome. This analysis allowed the retrieval of 417 ORFs containing MSE motifs in their promoters. These were mapped to GO terms and grouped according to Biological Process, Molecular Function or Cellular Component (Figure 5). Results showed that most ORFs with MSE elements (with over 10% and excluding the unknowns) belong to cell transport regulation, organelle organization, response to stress/chemical and RNA metabolic processes. Also, these ORFs are mostly related with enzymes with hydrolase or transferase activity which in addition to the cytoplasm and nucleus, many are located in cell membranes and mitochondria (Figure 5).

### C. parapsilosis *strains lacking* NDT80 *are more resistant to macrophage attack and impair macrophage viability*

**Figure 6.**
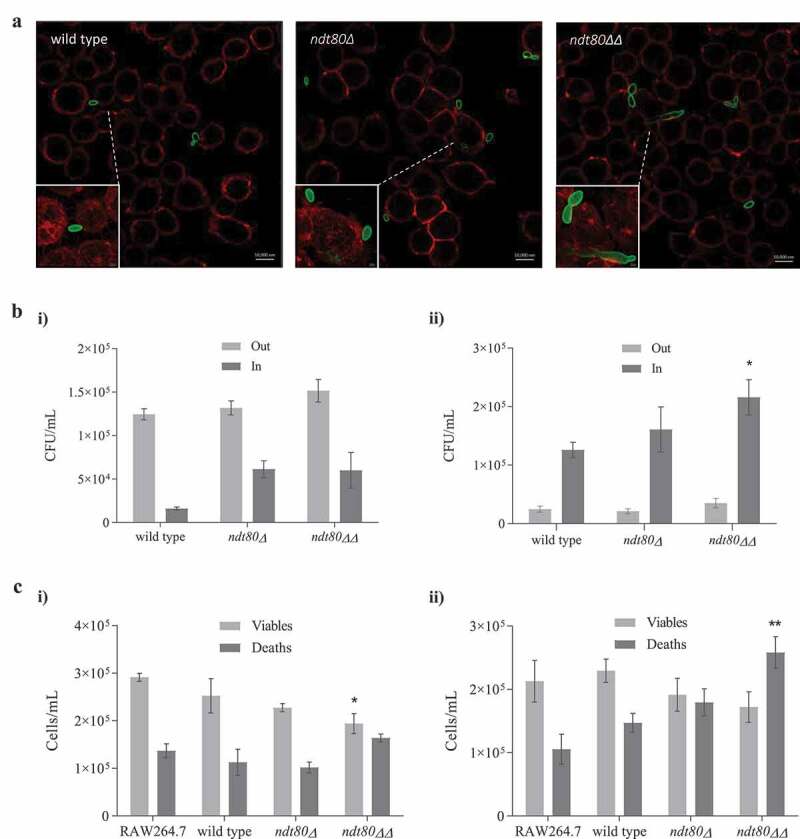
Interaction of *C. parapsilosis NDT80* deletion strains with RAW264.7 macrophage cells. (a) Representative confocal microscopy images of RAW264.7 macrophages and wild-type, *ndt80∆* and *ndt80∆∆* strains after 10 min of interaction at MOI of 1:1; scale bar represents 10 μm. Cells are distinguished through their different fluorescence staining with WGA (red macrophages) and Alexa Fluor 488 labeled anti-Candida antibody (green yeasts). Small boxes correspond to fluorescent projection details, highlighting mutant yeasts more adherent and internalized by macrophages (“tridimensional” images with fading green staining as indicator of phagocytosis and inclusion inside macrophages), when compared with wild-type. (b) Viable *C. parapsilosis* counts after i) 30 min and ii) 3 h interaction with macrophages at MOI of 1:1. Viable counts were performed using a CFU assay of co-culture supernatants (yeasts not internalized or adherent) and of lysed macrophage cells (phagocyted/internalized yeasts). (c) Viable and dead macrophage counts after i) 30 min and ii) 3 h interaction with *C. parapsilosis* strains at MOI of 1:1. Macrophage counts were performed after Trypan Blue exclusion test of cell viability. *p < 0.05 and **p < 0.01 wild-type or RAW264.7 macrophages control groups

The capacity of fungal cells to resist to macrophage-mediated killing contributes to its pathogenicity [36–38]. We conducted a phagocytic assay using the murine macrophage cell line RAW264.7 in order to determine the impact resulting from *NDT80* deletion upon phagocytic cells response. The interaction between macrophages and *C. parapsilosis* cells begins as early as 10 min (Figure 6(a)). However, while *C. parapsilosis* wild-type cells hardly interact, at the same time point a higher number of *ndt80ΔΔ* cells are attached to macrophages with clear signs of internalization, as indicated by the tridimensional green staining fading (Figure 6(a)); the *ndt80Δ* cells showed a intermediate behavior. Clearly, mutant strains exhibited a more effective adherence and internalization profile soon after 27 min of co-culturing (Movie S1), while this process is more lagging for the wild-type macrophage interaction; after 30 min of interaction, most of the *C. parapsilosis* cells were outside of the macrophages, adherent or not (Figure 6(b), i). Following 3 h of interaction, wild-type and both mutant strains were mostly internalized; notably, the number of *ndt80ΔΔ* mutant cells inside macrophages was statistically higher versus the two other cell types (Figure 6(b), ii).

Macrophage viability decreased along the assay (Figure 6(c), i and ii). Macrophage challenge with *ndt80ΔΔ* mutant cells, caused a significant reduction of the number of viable macrophages soon after 30 min (Figure 6(c), i). Following 3 h of co-culture, an increase of lysed macrophages was observed with all the strains assessed; however, this result was statistically significant in the case of *ndt80ΔΔ* strain (Figure 6(c), ii).

## Discussion

While molecular mechanisms are well characterized in *C. albicans*, several studies addressing the regulatory networks of non-*albicans* species, like *C. glabrata* and *C. parapsilosis*, demonstrate a significant difference in the evolutionary adaptation of such yeasts to the human host [19,39]. Although the available knowledge regarding the expression of *C. parapsilosis* virulence attributes is still somewhat limited, this species displays many biological features that are presumed to be directly related to its environmental colonization and pathogenicity, such as enhanced adherence and biofilm development on abiotic surfaces.

Adhesion, morphogenetic variations and biofilm formation are virulence attributes clearly depicted for *C. albicans* [40,41] and are intimately related to each other. Filamentous growth is closely related to the expression of surface proteins, such as Als1, Als3 and the hyphal-specific protein, Hwp1. In turn, these proteins play relevant roles in cell-cell and cell-surface adhesion and are required for biofilm formation as contact mediators that promote further biomass accumulation and enhance biofilm resilience [14,15]. Ndt80 was identified as one of the many regulators of filamentous growth by binding to promoters of genes encoding cell wall components (e.g. *ALS3* and *HWP1*), being required for their normal expression [42]. Thus, deletion of *NDT80* reduces *C. albicans* virulence *in vivo*, by blocking yeast to hyphal transition, as well as the expression of genes involved in the filamentous transcriptional program [42].

Surprisingly, and opposing to what was described for *C. albicans*, the disruption of *C. parapsilosis NDT80* gene triggers two noticeable phenotypic changes: morphogenesis in a spontaneous and constitutive manner (Figure 2), and prompted adhesion, both cell to cell and to abiotic surfaces, but also to murine macrophages (Figure 3 and Figure 6, respectively). Despite the scarce knowledge on *C. parapsilosis* adhesion mediators, we demonstrate that *ndt80* mutants adhesion is conferred by *ALS7* (*CPAR2*_*404800*), whose expression is extraordinary increased. This adhesin was previously identified as a mediator of *C. parapsilosis* adhesion to human buccal epithelial cells [31]. Although only 0.5% of the ORFs related with cell adhesion contain putative recognition sites for Ndt80, *ALS7* and *ALS3* are included in this group.

According to our findings Ndt80 can have a dual role in yeast to pseudohyphae transition: on one hand, by impairing the expression of *UME6* and *CPH2*, described as inducers of yeast to pseudohyphae transition [19]; on the other hand, by acting as an activator of Czf1 and Efg1 [19,35], two known transcription factors regulating phenotypic switching and filamentous growth in *C. albicans*. Other genes like *OCH1*, the orthologs of *C. albicans CWH41* and *STP3* are also involved in *C. parapsilosis* phenotypic switching, as positive and negative regulators, respectively [32,36]. We found that Ndt80 has no impact upon the expression of *OCH1* and the ortholog of *C. albicans STP3*; interestingly, the ortholog *C. albicans CWH41* expression doubles in *ndt80ΔΔ* mutant, suggesting that this gene could be a target for Ndt80, which putatively represses the expression of this pseudohyphae formation factor.

Ndt80 is also part of a network of six transcription factors (Bcr1, Efg1, Tec1, Rob1, Bgr1, and Ndt80) responsible for the regulation of *C. albicans* biofilm development [22]. In this species, *NDT80* deletion significantly compromises biofilm formation either *in vitro* or *in vivo* models [22]. Conversely, we found that deletion of *C. parapsilosis NDT80* gene promotes biofilm growth *in vitro*, suggesting that this transcription factor is acting as a repressor of genes involved in such process. Other biofilm regulators, acting as repressors and activators in a circuit system were already previously identified in *C. albicans* and *C. parapsilosis* [19]. Efg1, Bcr1, and Ace2 play similar roles regarding biofilm development in both species, while Cph2, Czf1, Gzf3, and Ume6 have major roles just in *C. parapsilosis* [19]. In *C. parapsilosis*, deletion of *CZF1, GZF3, UME6,* and *CPH2* was associated with a reduced biofilm formation ability. Although Ndt80 was not identified as a component of *C. parapsilosis* regulatory network due to the inherent growth defects [19], we analyzed the promoter sequences of all the biofilm transcription factors described by Holland *et al*. [19] for the presence of Ndt80 MSE motifs and identified putative recognition sites in all of the genes tested. The gene expression profile analysis of *ndt80ΔΔ* mutant revealed an approximately 36-fold, 13-fold, 4-fold and 3-fold upregulation of *MKC1, UME6, CPH2* and *ACE2*, respectively, while other genes also described to be required for biofilm formation, such as *GZF3* and *CZF1*, were demonstrated to be downregulated. These findings strongly suggest the role of Ndt80 as a negative regulator of *MKC1, UME6, CPH2* and *ACE2* expression and as an activator of *GZF3* and *CZF1* expression. Thus, in Ndt80 absence, and despite *GZF3* and *CZF1* genes exhibiting a reduced expression, the upregulation of *MKC1, UME6, CPH2* and *ACE2* genes occurs and biofilm development is promoted (Figure 4). *RHR2* was also considered to be involved in biofilm development by *C. parapsilosis*, as its expression was increased during biofilm formation [19]. Nevertheless, in *ndt80ΔΔ* mutant characterized by enhanced biofilm production, *RHR2* gene is downregulated probably denoting the lack of Ndt80 regulation as an activator.

The virulence-related phenotypes exhibited by *ndt80ΔΔ* mutant led us to explore its interaction with immune system cells. The ability to switch from yeast to a filamentous form is a key factor that allows successful phagocytosis evasion of *C. albicans* [43]. In the case of *C. parapsilosis*, several studies have elucidated distinct virulence traits of this species that could modulate the mechanism by which phagocytosis and the immune response proceed [44–46]. We found, in our *in vitro* infection assays a prompter interaction of both mutants with the macrophage cells in comparison to the wild-type strain. This finding is also in accordance with results obtained with the adhesion assays to abiotic surfaces and to other yeast cells.

Toth *et al*. [37] using other host cell models (J774.1 murine macrophage cell line and human peripheral blood mononuclear cells) described that the length of *C. parapsilosis* pseudohyphae did not correlate with the engulfment time. In our assays, after 3 h of co-culturing, only the *ndt80ΔΔ* mutant induced a significantly increase of macrophage killing with concomitant higher yeast viability, while neither the wild-type nor the *ndt80Δ* mutant promoted significant damage of the macrophage cells. These results show that the phenotype prompted by *NDT80* knockout results in a more virulent *C. parapsilosis* strains, more resistant to macrophage attack, associated with a decrease of macrophage cytoplasmic membrane integrity and a concomitant increase of macrophage cell death. Virulence features are not exclusively related to the constitutive pseudohyphal form; notably, the promoted expression of *ALS7* and *MKC1* transcripts (factors essential to cell wall integrity and remodeling) [47,48] provides a strong evidence of alterations of cell wall concerning composition and architecture in the *ndt80ΔΔ* mutant, with impact upon adhesion and recognition by immune system cells [49].

In fungi, *NDT80*-like genes recognize the conserved DNA-binding domain motif, MSE, through an Ig fold. As other members of the Ig-fold family of transcription factors, such as p53 or NFAR from mammals, *NDT80*-like genes share a similar regulation mechanism [50]. However, the number and attributable functions of *NDT80*-like genes are divergent among fungal species and even within species [34]. These disparities range from *NDT80* absence, as seen in *Schizosaccharomyces pombe*, to a family of six members, as seen in *Fusarium oxysporum*. While in *Saccharomyces cerevisiae, NDT80* single gene functions as a master regulator of meiosis process and sporulation [51], in other fungal species possessing several paralogous of *NDT80*-like genes the unraveling of its function and regulation mechanism is laborious and far from being obtained. NdtA and XprG are two of the Ndt80-like proteins in the filamentous fungal species *Aspergillus nidulans*. The former has a high homology with Ndt80 and like in *S. cerevisiae*, it is crucial for sexual reproduction. The later, under carbon starvation, regulates positively fungal response by controlling its extracellular proteases, mycotoxin, and penicillin expression, which could result in autolysis, hyphal fragmentation and ultimately in cell death [52]. *Neurospora crassa* possesses three Ndt80-like proteins, Vib-1, Ncu04729 and Fsd-1. Vib1, closely related to XprG, is an activator of extracellular protease production and is also associated with apoptosis [53]; Fsd1 (more similar to NdtA) together with Vib-1, is involved in the female sexual structure formation, but no one is required for meiosis. So far, NCU04729 gene deletion has no effect upon phenotype, which impairs the understanding of its function. In the CTG clade, *C. albicans* has three *NDT80*-like DNA-binding domain genes, *NDT80, RON1* and *REP1* [54]. These Ndt80-like transcription factors seem to be functionally independent from each other. Rep1 was found to be a regulator of the drug efflux pump *MDR1* and is required for yeast growth on presence of N-acetylglucosamine (GlcNAc) and galactose. Ron1 is associated with GlcNAc regulation signaling.

Notably, Ndt80 was identified as a morphogenesis and biofilm regulator, in *C. albicans* and *C. parapsilosis*, although it diverged to opposite functional roles. Our study highlights the importance of Ndt80 on the complex regulation of *C. parapsilosis* virulence attributes, as a major repressor.

## Supplementary Material

Supplemental Material
