# Supplementary figures and images for: The transcription factor Ndt80 is a repressor of Candida parapsilosis virulence attributes
Source: Virulence. 2021 Feb 4;12(1):601–14. doi: 10.1080/21505594.2021.1878743 (PMC7872087; doi:10.1080/21505594.2021.1878743)

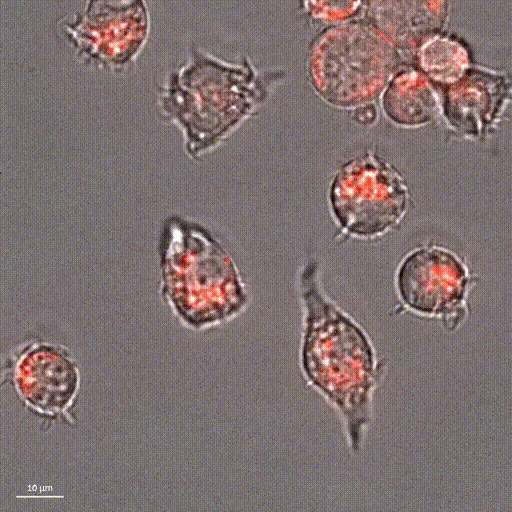

Supplement: Supplemental Material [file KVIR_A_1878743_SM0072.zip › supplement/Supplementary_MovieS1_a.gif]

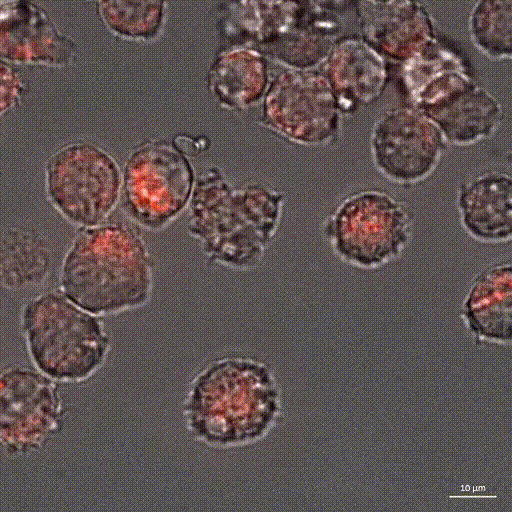

Supplement: Supplemental Material [file KVIR_A_1878743_SM0072.zip › supplement/Supplementary_MovieS1_b.gif]

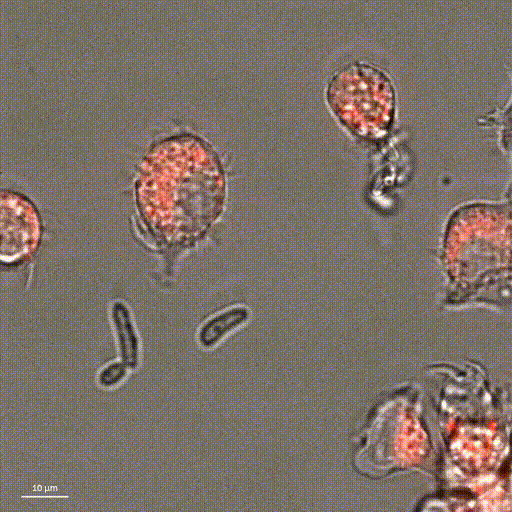

Supplement: Supplemental Material [file KVIR_A_1878743_SM0072.zip › supplement/Supplementary_MovieS1_c.gif]
